# Postoperative morbidity in patients with chronic viral hepatitis undergoing cardiac surgery: a retrospective study

**DOI:** 10.1186/1749-8090-8-S1-P68

**Published:** 2013-09-11

**Authors:** WC Hsieh, PC Chen, G Gradinariu, G Tinica

**Affiliations:** 1 Institute of Cardiovascular Disease, Grigore T. Popa University of Medicine and Pharmacy, Iasi, Romania; 2 Institute of Cardiovascular Disease, Iasi, Romania

## Background

Current cardiac risk scores such as EUROSCORE II and STS-Score do not take liver dysfunction into account despite the importance of liver function with respect to postoperative complications. This retrospective study attempts to assess the correlation of viral hepatitis with postoperative complications.

## Methods

We studied 103 patients with documented chronic viral hepatitis who had cardiac surgery in our institution between 2001-2012. These subjects were evaluated for preoperative liver dysfunction with the Model for End Stage Liver Disease (MELD) Score. In subjects who were on anticoagulants, a modified score (MELD-XI) was used. Other comorbidities that were considered were diabetes, dyslipidemia, obesity, hypertension, COPD, history of stroke and smoking status.

## Results

Mean age was 58.4±11.04 years; 62.1% of subjects were male. 66% of subjects had hypertension, 14.3% diabetes mellitus, 30% dyslipidemia, 23.3% were obese, 40.7% of subjects were smokers and 10.6% had a previous stroke. 40.7% of patients had chronic B hepatitis, 57.2% had chronic C hepatitis, while 1.9% had both. All patients underwent elective surgery; 39.8% had CABG, 25.2% had aortic valve replacement, 14.5% had mitral valve surgery and 20.3% had combined interventions. The average MELD score was 11.34±5.93 which corresponds to a mild to moderate liver dysfunction. Average EuroSCORE II was 2.07±1.62. Despite the low EuroSCORE II values, 10.6% of patients required prolonged mechanical ventilation (>24h) and 74.7% required inotropic support. Arrhythmias were seen postoperatively in 55.3% of cases, the most common being atrial fibrillation. 6.7% of patients required reinterventions, mainly for bleeding.

## Conclusions

The rate of complications in this group was higher than predicted by the EuroSCORE II and STS database, which underlines the fact that liver dysfunction associated with chronic viral hepatitis, represents an additional risk factor which should be assessed preoperatively.

